# Validity of the Ajinomoto Group Nutrient Profiling System Against Two 24 h Urinary Excretions of Sodium, Potassium and Protein in Japanese Adults

**DOI:** 10.3390/nu18101623

**Published:** 2026-05-20

**Authors:** Hiroko Jinzu, Sachi Nii, Keishiro Arima, Yuki Nakayama, Chie Furuta, Naoki Hayashi, Ryoko Tajima, Keiko Asakura, Shizuko Masayasu, Satoshi Sasaki, Kentaro Murakami, Hitomi Okubo

**Affiliations:** 1 Institute of Food Sciences and Technologies, Ajinomoto Co., Inc., Kawasaki 210-8681, Kanagawa, Japan; hiroko.jinzu.n4a@asv.ajinomoto.com (H.J.); sachi.nii.pj8@asv.ajinomoto.com (S.N.); keishiro.arima.cr6@asv.ajinomoto.com (K.A.); yuki.nakayama01.vf6@asv.ajinomoto.com (Y.N.); chie.furuta.cj3@asv.ajinomoto.com (C.F.); 2 Department of Nutritional Epidemiology and Behavioural Nutrition, Graduate School of Medicine, The University of Tokyo, Tokyo 113-0033, Japan; naokihayashi@g.ecc.u-tokyo.ac.jp (N.H.); ryktajima@m.u-tokyo.ac.jp (R.T.); 3 Department of Preventive Medicine, School of Medicine, Toho University, Tokyo 143-8540, Japan; keiko.asakura@med.toho-u.ac.jp; 4 Ikurien-Naka, Naka 311-0105, Ibaraki, Japan; sizuko-masa@themis.ocn.ne.jp; 5 Department of Social and Preventive Epidemiology, School of Public Health, Graduate School of Medicine, The University of Tokyo, Tokyo 113-0033, Japan; stssasak@m.u-tokyo.ac.jp (S.S.); kenmrkm@m.u-tokyo.ac.jp (K.M.)

**Keywords:** nutrient profiling system, dietary recommendations, nutritional value, 24 h urine collection, Japanese meals, validity

## Abstract

Background/Objectives: Nutrient profiling models are widely used to support healthier food choices, but their applicability may be limited in dietary cultures with multi-dish meals and high consumption of minimally processed foods. This study extended the Ajinomoto Group Nutrient Profiling System (ANPS), originally developed for dish- and meal-level assessment, to evaluate overall quality of daily intake (ANPS-Day) based on four components (protein, vegetables, saturated fatty acids [SFAs], and sodium), and examined its criterion-related validity using 24 h urinary biomarkers. Methods: A total of 324 healthy Japanese adults aged 20–69 years completed four-day semi-weighed dietary records and two non-consecutive 24 h urine collections. Urinary sodium, potassium and urea nitrogen were measured. Associations were examined using age- and sex-adjusted Spearman correlation coefficients and trend analyses. Results: The crude ANPS-Day score showed weak and inconsistent correlations with urinary biomarkers. In contrast, the energy-adjusted ANPS-Day score was positively correlated with estimated potassium intake (r = 0.25) and inversely correlated with the urinary sodium-to-potassium (Na/K) ratio (r = −0.24). In quartile analyses, higher energy-adjusted ANPS-Day scores were associated with higher protein and potassium intakes and with a lower Na/K ratio (all *p* for trend ≤ 0.001). In component analysis, vegetable points were positively associated with potassium intake, whereas sodium points were inversely associated with estimated sodium intake and the Na/K ratio. SFA points were not associated with urinary biomarkers. Conclusions: The energy-adjusted ANPS-Day score showed modest but biologically plausible associations with urinary biomarkers, providing partial evidence of criterion-related validity in assessing diet quality in multi-dish dietary settings.

## 1. Introduction

Nutrient profiling systems, operationalized through nutrient profiling models (NPMs), are widely used as public health tools to support healthier food choices by classifying or scoring foods according to their nutritional composition and potential health impact [1,2]. Most existing NPMs were developed in Western countries and are primarily designed to evaluate individual food items, reflecting dietary patterns characterized by frequent consumption of processed foods [3]. Many food-based NPMs, therefore, assess foods individually as discrete units of evaluation. Although this framework is appropriate where foods are commonly consumed independently, it may not fully represent dietary structure in cultures characterized by multi-dish meal patterns.

In many Asian settings, and in Japan in particular, meals consist of combinations of staple foods, main dishes and side dishes, with substantial contributions from minimally processed ingredients [3,4,5,6]. In such settings, overall diet quality is determined not only by the nutrient profile of individual foods but also by the balance and combination of dishes consumed within and across meals. Consequently, food-based NPMs that assess items in isolation may not adequately capture whole-diet quality in multi-dish dietary cultures, highlighting the need for profiling approaches capable of evaluating cooked dishes, complete meals and aggregated daily intake [7,8,9].

In response to this need, the Ajinomoto Group Nutrient Profiling System (ANPS) was developed to evaluate the nutritional quality of individual dishes (ANPS-Dish) and meals (ANPS-Meal), incorporating four components—protein, vegetables, saturated fatty acids (SFAs), and sodium—selected for their relevance to nutritional adequacy and public health nutrition priorities in the Japanese population [10,11]. These include persistently high sodium intake, increasing intake of SFAs with dietary westernization, and the need to promote adequate vegetable and protein intake in aging societies. Although convergent validity of ANPS-Dish and ANPS-Meal has been demonstrated against existing nutrient profiling and diet quality indices [10,11], validation has relied primarily on nutrient composition data derived from existing recipe databases. Criterion-related validity against objective biological measures has not been established.

Establishing validity using recovery biomarkers is particularly important for NPMs derived from self-reported dietary data, which are subject to measurement error and reporting bias [12]. Twenty-four-hour urinary biomarkers provide objective estimates of sodium, potassium, and protein intake [13,14,15]. Moreover, the urinary sodium-to-potassium ratio (Na/K) has been shown to be more strongly associated with blood pressure and cardiovascular disease risk than sodium or potassium alone [16,17,18,19,20,21], supporting its relevance as an integrated marker of diet quality. Although associations between diet quality indices and urinary biomarkers have been reported [22,23,24], evidence directly linking dish- or meal-based NPM scores to biologically assessed nutrient exposure at the whole-diet level remains limited. Moreover, it remains unclear whether an NPM originally designed for dish- and meal-level assessment can be meaningfully extended to daily dietary intake and reflect biologically assessed nutrient exposure. Given that recovery biomarkers are available only for selected nutrients, validation efforts must necessarily focus on components that can be assessed using objective biological measures.

Therefore, the present study aimed to extend the ANPS model to evaluate the overall quality of the daily diet (ANPS-Day) and to examine its association with 24 h urinary biomarkers of sodium, potassium and protein excretion, as well as the urinary Na/K ratio, in healthy Japanese adults.

## 2. Materials and Methods

### 2.1. Data Source and Study Participants

This validation study used data from a multi-center cross-sectional survey conducted between February and March 2013, which aimed to assess sodium and potassium intake among apparently healthy Japanese adults aged 20–69 years. The survey was conducted in 20 areas across Japan, selected to ensure geographical representation and logistical feasibility. A detailed description of the survey has been published elsewhere [25,26]. Briefly, 199 dietitians working in welfare facilities across the 20 survey areas served as research collaborators. Each research dietitian recruited colleagues, including clerical staff, care workers, medical assistants, and cooking assistants, as study participants. Recruitment targeted 40 participants per area, comprising 4 men and 4 women in each of five 10-year age groups (20–29 to 60–69 years). When insufficient numbers were available in the oldest age group, neighbors or acquaintances aged 60 and over were additionally included. Participants were volunteers and were not randomly selected.

Exclusion criteria were as follows: (i) certified dietary or medical professionals (e.g., dietitians, nurses or physicians); (ii) individuals who had not resided in the study prefecture or an adjacent prefecture before 1 August 2012; (iii) individuals undergoing a dietary therapy prescribed by a medical professional at the time of the study or within the past year; (iv) pregnant or lactating women; (v) individuals with a history of educational hospitalization for diabetes management.

The present study is a secondary analysis of this existing dataset, and the sample size was determined by the design of the original study [26]. Therefore, no a priori sample size calculation was performed specifically for the present analysis. Of the 791 study participants (395 men and 396 women), approximately half (400 participants) were additionally asked to complete dietary records according to the original study design, which incorporated a subsampling strategy to balance feasibility and data quality. For these participants, information on age, educational background, smoking status and physical activity level was collected using questionnaires. Anthropometric measurements were obtained using standardized procedures.

### 2.2. Dietary Records

Dietary data were collected using semi-weighed dietary records (DRs) on four non-consecutive days [26]. Participants were instructed to record all foods and beverages consumed on three working days and one non-working day, avoiding days immediately before and after night shifts. Each participant was provided with a digital kitchen scale (KD-812WH, Tanita, Tokyo, Japan), measuring utensils, and standardized recording forms, along with detailed instructions from the research dietitian. The recording form was structured based on a typical Japanese meal pattern consisting of breakfast, lunch, dinner, and snacks. Participants recorded the name of dishes and food items, as well as their actual or estimated amounts (weighed with a measuring instrument or by number of items), for each eating occasion. When weighing was impractical (e.g., dining out), participants recorded details like the restaurant name, dish name, and whether any leftovers remained. All completed records were reviewed by a research dietitian to clarify any missing or ambiguous information and then reconfirmed by two research dietitians at the central office of the study.

Foods were coded according to the Standard Tables of Food Composition in Japan 2015-(Seventh Revised Edition) [27]. Nutrient intakes from foods and beverages, excluding dietary supplements, were calculated. For each participant, the mean daily intakes of energy, protein, SFAs, sodium and vegetables across four days were computed for subsequent ANPS calculation.

### 2.3. Calculation of the Ajinomoto Group Nutrient Profiling System for Japanese Overall Diet per Day

ANPS comprises two previously developed NPMs, ANPS-Dish and ANPS-Meal, which evaluate the nutritional quality of the Japanese diet at the dish and meal levels, respectively [10,11]. The ANPS-Dish evaluates the nutritional quality of 13 dish categories. For example, one main-dish category (subcategory 6) includes dishes with total ingredients ≥ 120 g and protein ≥ 6 g. In contrast, the ANPS-Meal evaluates the nutritional quality of diverse single meals composed of combinations of staple foods, main dishes, soups, and side dishes. Both models assign scores ranging from 0 to 100, based on the adequacy of protein, vegetables, SFAs and sodium content in a single dish or a meal relative to target values, with higher score indicating better nutritional quality. The overall ANPS score is calculated as the sum of the protein, vegetable, SFAs, and sodium points, each ranging from 0 to 10, multiplied by 2.5.

In the present study, we extended the ANPS model to evaluate the total daily intake, referred to as ANPS-Day. The scoring for ANPS-Day was based on the daily target values established during the development of the ANPS-Dish and ANPS-Meal, which were set at 2000 kcal/d for energy, 66.0 g/d of protein, 350.0 g/d of vegetables, 22.22 g/d for SFAs (equivalent to 10% energy intake), and 2756 mg/d of sodium (equivalent to 7.0 g/d of salt) [10,11]. For the encouraged intake components, namely protein and vegetables, 1 point was deducted for every 10% decrease from the target values. For limited intake components, SFAs and sodium, 1 point or 0.5 points were deducted for every 10% increase from the target value, respectively (Table 1).

Because the ANPS-Day score may be influenced by individual differences in total energy intake, we also calculated energy-adjusted ANPS-Day scores using the density method standardized to 2000 kcal/d. This approach was used to account for inter-individual variation in energy intake and to facilitate evaluation of diet quality independent of total energy intake.

### 2.4. 24 h Urine Collection

Urine samples were collected on two non-consecutive days, one before and one after the four-day DRs, to minimize participant burden and enhance data quality. Detailed description of the urine collection has been reported elsewhere [25]. Participants recorded the start and end times of each collection period, ensuring complete sample collection. In case of missed urine, participants were instructed to estimate the volume and report it. All urine samples were stored under conditions avoiding extreme temperature and subsequently analyzed for concentrations of sodium, potassium, urea nitrogen (Urea-N), and creatinine at LSI Medience Corporation (Tokyo, Japan). Sodium and potassium concentrations were measured using the electrode method with a JCA-BM6050 clinical chemistry analyzer (JEOL Limited, Tokyo, Japan). Urea-N concentration was measured via the urease LEDH method using Iatro-LQ UN rate (A) II assay (LSI Medience Corporation), and creatinine concentration was measured via the enzyme method using the Iatro-LQ CRE(A) II assay (LSI Medience Corporation).

### 2.5. Calculation of Biomarkers

The 24 h urinary excretion of sodium, potassium and Urea-N was calculated by multiplying the measured concentrations by the adjusted 24 h urinary volume, which accounted for the recorded collection duration and any missing urine volumes [28]. The validity of this adjustment method was confirmed through the para-aminobenzoic acid check method [28].

Intakes of sodium, potassium and protein were estimated from the 24 h urinary excretion according to previously reported methods [29]. Dietary sodium and potassium intakes (mg/d) were calculated by dividing the 24 h urinary sodium and potassium excretion by the urinary excretion rates (0.86 and 0.77, respectively) [13,15]. Dietary protein intake (g/d) was calculated by first dividing the 24 h urinary UN excretion by the ratio of urinary urea nitrogen excretion to urinary total nitrogen excretion (0.85) and the urinary excretion rate (0.81) and then multiplying by the nitrogen-to-protein conversion factor (6.25) [14].

Estimated dietary intakes were energy-adjusted using the density method. Each nutrient intake was divided by the individual estimated energy requirement (EER) and multiplied by 2000 to express values per 2000 kcal/d. The EER was calculated using the sex-, age-, weight-, height-, and physical activity-specific equations provided in the US Dietary Reference Intakes [30].

The urinary Na/K ratio was calculated by dividing the 24 h urinary sodium excretion (mmol/d) by the 24 h urinary potassium excretion (mmol/d).

### 2.6. Statistical Analysis

A total of 392 participants (196 men and 196 women) completed both the 4 d DRs and two 24 h urine collections. The completeness of urine collection was assessed using the ratio of observed-to-expected creatinine excretion, based on sex-specific expected values, as previously described [29]. Participants with a ratio of <60% or >140% were excluded (*n* = 66) [25,28,31]. Two additional participants with insufficient intervals (<3 d) between 24 h urine collections were excluded [25], resulting in a final analytic sample of 324 participants (161 men and 163 women).

Descriptive statistics for participant characteristics and dietary data were presented as means (SDs) for continuous variables and number (%) of participants for categorical variables. Associations of ANPS-Day score and its component points with urinary biomarkers were examined using partial Spearman correlation coefficients adjusted for sex and age. For the crude ANPS-Day score, associations were examined with 24 h urinary excretion values, whereas for the energy-adjusted ANPS-Day score, associations were examined with biomarker-derived estimates of dietary intake. Participants were divided into quartiles according to the ANPS-Day score or each component point. Correlation analyses were used to evaluate overall associations between continuous variables, whereas quartile analyses were conducted to illustrate potential dose–response patterns and provide more interpretable comparisons of biomarker levels across score categories. The two approaches were used in a complementary manner. The magnitude of the observed correlations was contextualized with reference to previously published biomarker validation studies of diet quality indices, in which correlations with recovery biomarkers are generally modest in magnitude [12]. To assess linear trends, the median value of each quartile was assigned and modeled as a continuous variable in multivariable regression analyses adjusted for age and sex. Although no corresponding urinary biomarker is available to directly assess SFA intake, the SFA point was analyzed in the same way as the other component points. Sensitivity analyses were performed using tertiles for components with skewed distributions to confirm the robustness of the results. In addition, sensitivity analyses applying Bonferroni correction were conducted for the primary correlation analyses between the energy-adjusted ANPS-Day score and urinary biomarkers. All statistical analyses were performed using R version 4.0.4. Statistical significance was defined as a two-sided *p*-value < 0.05 unless otherwise specified.

## 3. Results

### 3.1. Participant Characteristics

Table 2 shows the basic characteristics of the participants. The mean age and body mass index (BMI) were 44.8 (SD 13.1) years and 23.1 (SD 3.3) kg/m^2^, respectively. Estimated daily intakes from 24 h urine collection were 4849 (SD 1549) mg/d for sodium, 2430 (SD 814) mg/d for potassium, and 76.8 (SD 18.1) g/d for protein, with a mean Na/K ratio of 4.0 (SD 1.2). The mean crude and energy-adjusted ANPS-Day scores were 77.7 (SD 7.4) and 80.2 (SD 6.7), respectively.

### 3.2. Associations Between the ANPS-Day Score and Urinary Biomarkers

Table 3 shows associations between the ANPS-Day score and urinary biomarkers. The crude ANPS-Day score showed weak and inconsistent correlations with urinary biomarkers. In contrast, the energy-adjusted ANPS-Day score was modestly correlated with estimated potassium intake (r = 0.25) and was inversely correlated with the Na/K ratio (r = −0.24). The positive association between the energy-adjusted ANPS-Day score and estimated potassium intake and the inverse association with the urinary Na/K ratio remained statistically significant after Bonferroni correction. In trend analysis, higher quartiles of crude ANPS-Day score were associated with higher urinary potassium excretion (*p* for trend = 0.04) and a lower Na/K ratio (*p* for trend = 0.02). For the energy-adjusted ANPS-Day score, higher quartiles were significantly associated with higher estimated potassium (*p* < 0.001) and protein intakes (*p* = 0.001), as well as a lower Na/K ratio (*p* < 0.001).

### 3.3. Associations Between ANPS-Day Components and Urinary Biomarkers

Table 4 summarizes associations between each component of the ANPS-Day and urinary biomarkers. For crude ANPS-Day components, protein point was positively correlated with urinary Urea-N excretion (r = 0.25), vegetable point was positively correlated with urinary potassium (r = 0.28) and Urea-N excretion (r = 0.20), and sodium points were inversely correlated with urinary sodium excretion (r = −0.31). Trend analysis showed that higher protein and vegetable points were associated with greater urinary potassium and Urea-N excretion, and higher vegetable points were additionally associated with a lower Na/K ratio. A higher sodium point was associated with lower urinary sodium, potassium, Urea-N excretion and a lower Na/K ratio. A higher SFA point was associated with lower Urea-N excretion.

Table 5 summarizes associations between the energy-adjusted ANPS-Day components and urinary biomarkers. For the energy-adjusted ANPS-Day components, correlation patterns were generally consistent with those observed for the crude components. The vegetable point was positively correlated with estimated potassium intake (r = 0.29), and sodium point was inversely correlated with estimated sodium intake (r = −0.26). The SFA point showed no clear correlation with the urinary biomarkers examined. In trend analysis, a higher protein point was significantly associated with higher estimated protein intake, corresponding to an approximate difference of 11.3 g/d between Q4 and Q1. Higher vegetable points were significantly associated with higher estimated intakes of sodium, potassium, and protein, as well as a lower Na/K ratio. Higher SFA points were significantly associated with greater estimated intake of sodium and potassium. In contrast, higher sodium points were significantly associated with lower estimated intakes of sodium and a lower Na/K ratio.

Sensitivity analyses using tertiles for protein and SFA points were conducted due to the unequal distribution across quartiles; the results were not materially altered.

## 4. Discussion

In this study, we examined the associations between ANPS-Day and 24 h urinary biomarkers of sodium, potassium, and protein intake in healthy Japanese adults to provide evidence regarding criterion-related validity. The energy-adjusted ANPS-Day score showed consistent and biologically plausible associations with higher potassium and protein intake and a lower Na/K ratio, whereas associations for the crude score were weaker and less consistent. Although the observed correlations were modest in magnitude, their directional consistency and biological plausibility provide supportive, albeit limited, evidence regarding the validity of the scoring framework. The magnitude of the observed correlations was broadly comparable to those reported in previous biomarker validation studies of diet quality indices and nutrient profiling systems, in which correlations with recovery biomarkers are generally modest in magnitude [12]. To our knowledge, this study is among the few to demonstrate that an NPM originally developed for dishes and meals can be extended to daily dietary intake and evaluated against objective biomarker data.

A key finding of this study is that associations between ANPS-Day and urinary biomarkers were more clearly observed for the energy-adjusted score than for the crude score. For the crude ANPS-Day score, correlations with urinary biomarkers were generally weak, although quartile-based trend analyses suggested some generally consistent directional patterns. These findings suggest that the underlying scoring structure remained interpretable even before energy adjustment. This discrepancy may reflect the influence of total energy intake. Crude dietary scores inevitably reflect not only diet quality but also total food intake, which varies substantially according to body size, physical activity, and energy requirements. Such variability may obscure associations with nutrient-related biomarkers. In contrast, energy adjustment using the density method attenuates the influence of total intake volume and highlights the relative balance of foods and nutrients within the diet. Because NPMs are conceptually intended to evaluate diet quality rather than quantity, the energy-adjusted ANPS-Day score should be regarded as the primary indicator in epidemiological interpretation.

The observed associations between ANPS-Day and the urinary Na/K ratio are particularly relevant from a public health perspective. Previous studies have consistently shown that healthier dietary patterns are associated with a lower urinary Na/K ratio and higher potassium intake [32,33]. The Na/K ratio has been reported to show stronger and more consistent associations with blood pressure than sodium or potassium intake alone [20,21]. Moreover, lower scores on comparable nutrient-based indices, such as Food Standards Agency Nutrient Profiling System underlying Nutri-Score and the Healthy Eating Index-2015, have been associated with elevated blood pressure and increased CVD risk [34,35,36,37,38,39]. Taken together, the present findings suggest that ANPS-Day reflects dietary characteristics relevant to cardiometabolic health, although its predictive validity for clinical outcomes requires confirmation in longitudinal studies.

Although several examined biomarkers were directly related to nutrients incorporated into the ANPS-Day score, demonstrating expected associations with objective recovery biomarkers remains an important step in evaluating criterion-related validity for a nutrient profiling system originally derived from self-reported dietary intake data. Vegetable, protein, and sodium points were each associated with urinary or estimated intakes of potassium, protein, and sodium, respectively, in directions consistent with the intended design of the ANPS model. Although the effect sizes were modest, the internal coherence across components strengthens confidence in the multidimensional scoring structure. Importantly, cross-associations were also observed. Higher vegetable points were associated not only with greater potassium intake but also with higher protein and sodium intake, while higher protein points were associated with greater estimated potassium intake. These patterns likely reflect the structure of traditional Japanese meals, which typically combine staple foods with protein-rich main dishes and vegetable-based side dishes, often seasoned with sodium-rich condiments such as soy sauce and miso [40,41,42].

These findings likely reflect a structural characteristic of traditional Japanese dietary patterns, in which vegetable-rich dishes are commonly consumed together with sodium-rich seasonings and cooking methods. Therefore, individuals with higher vegetable point scores may simultaneously have higher sodium exposure. Rather than indicating inconsistency in the scoring system, this finding highlights the importance of multidimensional nutrient profiling approaches that simultaneously evaluate both favorable and unfavorable nutritional attributes when assessing overall diet quality in Japanese dietary settings. In this context, the observed paradox does not undermine the validity of the ANPS-Day score but rather illustrates the practical importance of evaluating multiple nutritional dimensions simultaneously in multi-dish dietary cultures.

A major strength of this study is the use of 24 h urine collections, which provide objective and reliable estimates of sodium, potassium, and protein intake. This enabled a robust evaluation of the criterion-related validity of ANPS-Day beyond self-reported dietary data alone. Nevertheless, several limitations should be acknowledged. First, the study population consisted of employees of social welfare facilities, which may limit generalizability due to potentially higher health consciousness than that of the general population. The relatively homogeneous and health-conscious nature of the sample may also have reduced variability in dietary exposure, potentially attenuating observable associations with urinary biomarkers. Accordingly, caution is warranted when generalizing these findings to the broader Japanese population. Second, the data used for analysis were collected in 2013, and dietary habits may have changed since then. However, the primary aim of the present study was to examine whether ANPS-Day scores are biologically associated with objective markers of sodium, potassium, and protein exposure, rather than to estimate current population dietary intake levels. The physiological relationships underlying these associations are unlikely to have been fundamentally altered by dietary shifts over time. In addition, sodium intake among Japanese adults remains persistently high and vegetable intake continues to fall below national targets [43], suggesting that the nutritional priorities underlying the ANPS scoring framework remain relevant under contemporary dietary conditions. Nevertheless, future validation studies using more contemporary datasets would be valuable. Third, biomarker coverage was limited. Although the ANPS model includes four components—protein, vegetable, SFAs, and sodium—only sodium, potassium (as an indicator related to vegetable-rich dietary patterns), and urea-N (as a proxy of protein intake) could be evaluated using 24 h urine collections. Because SFA intake cannot be directly assessed using urinary biomarkers, the present findings should be interpreted as partial validation of the ANPS-Day system rather than validation of all score components. Future studies should incorporate alternative objective measures, such as circulating fatty acid composition [44], to further evaluate this component. Fourth, the component-level analyses should be interpreted cautiously because multiple statistical comparisons were performed and these analyses were exploratory in nature. Fifth, no formal sample size calculation was performed specifically for the present analysis because this study was based on an existing dataset. Finally, the relatively short duration of dietary recording and urine collection may not fully capture habitual intake, although longer assessment periods may reduce compliance and alter usual dietary behavior.

## 5. Conclusions

This cross-sectional study showed modest but biologically plausible associations between ANPS-Day and 24 h urinary biomarkers, providing partial evidence supporting its criterion-related validity as a culturally adapted tool for evaluating daily diet quality. The clearer associations observed for the energy-adjusted score highlight the importance of accounting for variation in total energy intake when applying NPMs in epidemiological settings. These findings provide empirical support for extending meal-based nutrient profiling approaches to whole-diet assessment in multi-dish dietary contexts, while underscoring the need for prospective studies to evaluate their predictive validity for health outcomes.

## Figures and Tables

**Table 1:** Scoring table of the Ajinomoto Group Nutrient Profiling System for Japanese Overall Diet per day (ANPS-Day).

	Protein	Vegetable	SFAs		Sodium
Points	(g)	(g)	(g)	Points	(mg)
10	≥66.0	≥350.0	≤22.22	10	≤2756
9	≥59.4	≥315.0	≤24.44	9.5	≤3031
8	≥52.8	≥280.0	≤26.66	9	≤3307
7	≥46.2	≥245.0	≤28.89	8.5	≤3583
6	≥39.6	≥210.0	≤31.11	8	≤3858
5	≥33.0	≥175.0	≤33.33	7.5	≤4134
4	≥26.4	≥140.0	≤35.55	7	≤4409
3	≥19.8	≥105.0	≤37.77	6.5	≤4685
2	≥13.2	≥70.0	≤40.00	6	≤4961
1	≥6.6	≥35.0	≤42.22	5.5	≤5236
0	<6.6	<35.0	>42.22	5	≤5512
				4.5	≤5787
				4	≤6063
				3.5	≤6339
				3	≤6614
				2.5	≤6890
				2	≤7165
				1.5	≤7441
				1	≤7717
				0.5	≤7992
				0	>7992

SFAs, saturated fatty acids.

**Table 2:** Basic characteristics of the participants among 324 Japanese men and women aged 20–69 years *.

Variable	*n*	%	Mean	SD
Sex				
Men	161	49.7		
Women	163	50.3		
Age group				
20–29	57	17.6		
30–39	70	21.6		
40–49	67	20.7		
50–59	63	19.4		
60–69	67	20.7		
Living area				
Hokkaido and Tohoku	42	13		
Kanto	66	20.4		
Hokuriku and Tokai	36	11.1		
Kinki	50	15.4		
Chugoku and Shikoku	66	20.4		
Kyushu and Okinawa	64	19.8		
Educational background				
Junior or senior high school	94	29		
Vocational school or junior college	113	34.9		
University or graduate school	116	35.8		
Others	1	0.3		
Smoking status				
Nonsmoker	188	58		
Past smoker	61	18.8		
Current smoker	75	23.1		
Energy intake from dietary records (kcal/d)			2138	472
24 h Urinary excretion				
Sodium (mg/d)			4340	1435
Potassium (mg/d)			1927	595
Urea-N (mg/d)			8856	2483
Na/K ratio (mmol/mmol)			4.0	1.2
Dietary intake estimated from 24 h Urinary excretion ^†^				
Sodium intake (mg/d)			4849	1549
Potassium intake (mg/d)			2430	814
Protein intake (g/d)			76.8	18.1
Crude ANPS-Day and each component point				
ANPS-Day (point/d)			77.7	7.4
Protein point (point/d)			9.0	1.1
Vegetable point (point/d)			6.1	2.2
SFAs point (point/d)			8.7	1.6
Sodium point (point/d)			7.3	1.7
Energy-adjusted ANPS-Day and each component point ^‡^				
ANPS-Day (point/d)			80.2	6.7
Protein point (point/d)			9.2	0.8
Vegetable point (point/d)			5.9	2.2
SFAs point (point/d)			9.3	1.0
Sodium point (point/d)			7.7	1.4

ANPS-Day, the Ajinomoto Group Nutrient Profiling System for Japanese Overall Diet per day; SFAs, saturated fatty acids; Urea-N, urea nitrogen; Na/K ratio, sodium-to-potassium ratio; SD, standard deviation. * The values were expressed as mean and SD; numbers and percentages. ^†^ Dietary intake estimated from 24 h urinary excretion was calculated as follows: sodium intake (mg/d) = 24 h urinary sodium excretion (mg/d)/0.86; potassium intake (mg/d) = 24 h urinary potassium excretion (mg/d)/0.77; and protein intake (g/d) = 24 h urinary urea-N (g/d)/(0.85 × 0.81) × 6.25. ^‡^ Energy-adjusted ANPS-Day and each component point were calculated using the density method standardized to 2000 kcal/d.

**Table 3:** Estimated dietary intake and 24 h urinary excretion according to quartile (Q) of ANPS-Day among 324 Japanese men and women.

Variable	Q1	Q2	Q3	Q4	*p* for Trend ^†^	*r* ^‡^
Crude ANPS-Day score						
*n*	79	83	76	86		
Median (IQR)	69.4 (65.2–70.9)	75.3 (74.1–76.9)	80.6 (79.5–81.6)	85.9 (84.1–88.4)		
24 h Urinary excretion *						
Sodium (mg/d)	4359 ± 1641	4456 ± 1309	4203 ± 1432	4329 ± 1364	0.61	0.02
Potassium (mg/d)	1841 ± 664	1856 ± 440	1930 ± 553	2074 ± 675	0.04	0.13
Urea-N (mg/d)	8932 ± 3091	8945 ± 1970	8579 ± 2400	8944 ± 2400	0.46	0.06
Na/K ratio (mmol/mmol)	4.2 ± 1.1	4.2 ± 1.4	3.9 ± 1.3	3.7 ± 1.1	0.02	−0.17
Energy-adjusted ANPS-Day score						
*n*	77	85	80	82		
Median (IQR)	72.8 (69.7–73.8)	78.1 (76.9–79.1)	83.0 (82.2–83.8)	87.5 (86.3–89.4)		
Dietary intake estimated from 24 h Urinary excretion *^§^						
Sodium (mg/d)	4441 ± 1527	4965 ± 1616	5057 ± 1432	4914 ± 1562	0.99	0.04
Potassium (mg/d)	2038 ± 623	2361 ± 682	2479 ± 776	2828 ± 949	<0.001	0.25
Protein (g/d)	68.4 ± 18.1	77.9 ± 14.6	78.1 ± 17.4	82.4 ± 19.6	0.001	0.18
24 h Urinary excretion						
Na/K ratio (mmol/mmol)	4.3 ± 1.1	4.2 ± 1.4	4.1 ± 1.3	3.5 ± 1.0	<0.001	−0.24

ANPS-Day, the Ajinomoto Group Nutrient Profiling System for Japanese Overall Diet per day; Q, quartile; Urea-N, urea nitrogen; Na/K ratio, sodium-to-potassium ratio; SD, standard deviation; IQR, inter-quartile range. * Values are presented as Mean ± SD. ^†^ Trend of association was examined using a multivariable linear regression model adjusted for age and sex, with the median value of the ANPS-Day score in each quartile modelled as a continuous variable. ^‡^ Partial Spearman correlation coefficients adjusted for age and sex were calculated. ^§^ Dietary intake estimated from 24 h urinary excretion was calculated as follows: sodium intake (mg/d) = 24 h urinary sodium excretion (mg/d)/0.86; potassium intake (mg/d) = 24 h urinary potassium excretion (mg/d)/0.77; and protein intake (g/d) = 24 h urinary urea-N (g/d)/(0.85 × 0.81) × 6.25.

**Table 4:** Estimated dietary intake and 24 h urinary excretion according to quartile (Q) of crude ANPS-Day components among 324 Japanese men and women.

Variable	Q1	Q2	Q3	Q4	*p* for Trend ^†^	*r* ^‡^
Protein point						
*n*	77	67	79	101		
Median (IQR)	7.8 (6.8–8.0)	8.8 (8.5–9.0)	9.5 (9.3–9.8)	10.0 (10.0)		
24 h Urinary excretion *						
Sodium (mg/d)	3812 ± 1236	4230 ± 1391	4663 ± 1566	4571 ± 1398	0.03	0.10
Potassium (mg/d)	1794 ± 517	1716 ± 522	1969 ± 527	2130 ± 675	0.01	0.11
Urea-N (mg/d)	7810 ± 2094	7873 ± 2133	9087 ± 2261	10,103 ± 2537	<0.001	0.25
Na/K ratio (mmol/mmol)	3.8 ± 1.2	4.3 ± 1.3	4.1 ± 1.2	3.9 ± 1.3	0.99	−0.03
Vegetable point						
*n*	73	86	77	88		
Median (IQR)	3.0 (2.5–3.8)	5.3 (4.8–5.8)	6.8 (6.5–7.0)	8.8 (8.3–9.5)		
24 h Urinary excretion *						
Sodium (mg/d)	3925 ± 1435	4436 ± 1356	4338 ± 1397	4603 ± 1491	0.02	0.18
Potassium (mg/d)	1676 ± 528	1901 ± 544	1883 ± 536	2206 ± 643	<0.001	0.28
Urea-N (mg/d)	8156 ± 2462	9029 ± 2551	8660 ± 2391	9458 ± 2380	0.001	0.20
Na/K ratio (mmol/mmol)	4.2 ± 1.3	4.1 ± 1.1	4.1 ± 1.5	3.7 ± 1.1	0.02	−0.14
SFAs point						
*n*	76	76	83	89		
Median (IQR)	6.5 (5.5–7.5)	8.5 (8.3–9.0)	9.5 (9.5–9.8)	10.0 (10.0)		
24 h Urinary excretion *						
Sodium (mg/d)	4375 ± 1338	4116 ± 1418	4451 ± 1543	4397 ± 1431	0.94	0.01
Potassium (mg/d)	1947 ± 568	1802 ± 583	2014 ± 628	1934 ± 589	0.70	−0.03
Urea-N (mg/d)	9510 ± 2622	8353 ± 2481	9139 ± 2422	8463 ± 2289	0.03	−0.14
Na/K ratio (mmol/mmol)	4.0 ± 1.0	4.1 ± 1.5	3.9 ± 1.2	4.1 ± 1.3	0.71	0.03
Sodium point						
*n*	81	76	86	81		
Median (IQR)	5.6 (4.5–6.0)	6.8 (6.6–7.0)	8.1 (7.6–8.4)	9.3 (9.0–9.6)		
24 h Urinary excretion *						
Sodium (mg/d)	5090 ± 1438	4544 ± 1412	4173 ± 1384	3580 ± 1060	<0.001	−0.31
Potassium (mg/d)	2109 ± 620	1970 ± 581	1864 ± 592	1770 ± 542	0.01	−0.15
Urea-N (mg/d)	9953 ± 2577	9093 ± 2206	8459 ± 2636	7961 ± 2028	0.003	−0.18
Na/K ratio (mmol/mmol)	4.3 ± 1.5	4.1 ± 1.0	4.0 ± 1.2	3.6 ± 1.2	0.002	−0.16

ANPS-Day, the Ajinomoto Group Nutrient Profiling System for Japanese Overall Diet per day; Q, quartile; SFAs, saturated fatty acids; Urea-N, urea nitrogen; Na/K ratio, sodium-to-potassium ratio; SD, standard deviation; IQR, inter-quartile range. * Values are presented as Mean ± SD. ^†^ Trend of association was examined using a multivariable linear regression model adjusted for age and sex, with the median value of the ANPS-Day score in each quartile modelled as a continuous variable. **^‡^** Partial Spearman correlation coefficients adjusted for age and sex were calculated.

**Table 5:** Estimated dietary intake and 24 h urinary excretion according to quartile (Q) of energy-adjusted ANPS-Day components among 324 Japanese men and women.

Variable	Q1	Q2	Q3	Q4	*p* for Trend ^†^	*r* ^‡^
Protein point						
*n*	58	97	54	115		
Median (IQR)	8.3 (7.8–8.5)	9.0 (8.8–9.3)	9.5 (9.5)	10.0 (9.8–10.0)		
Dietary intake estimated from 24 h Urinary excretion *^§^						
Sodium (mg/d)	4436 ± 1153	4882 ± 1574	4808 ± 1617	5056 ± 1646	0.42	0.06
Potassium (mg/d)	2201 ± 650	2317 ± 713	2422 ± 761	2647 ± 942	0.06	0.10
Protein (g/d)	70.8 ± 16.1	74.5 ± 14.8	76.4 ± 20.3	82.1 ± 19.4	0.002	0.17
24 h Urinary excretion						
Na/K ratio (mmol/mmol)	4.1 ± 1.1	4.2 ± 1.3	4.0 ± 1.3	3.8 ± 1.2	0.33	−0.09
Vegetable point						
*n*	68	88	84	84		
Median (IQR)	3.1 (2.5–3.5)	5.0 (4.3–5.5)	6.5 (6.0–7.0)	8.8 (8.0–9.3)		
Dietary intake estimated from 24 h Urinary excretion *^§^						
Sodium (mg/d)	4245 ± 1270	4797 ± 1575	4892 ± 1456	5370 ± 1658	0.01	0.16
Potassium (mg/d)	1977 ± 575	2356 ± 760	2430 ± 702	2889 ± 914	<0.001	0.29
Protein (g/d)	69.4 ± 19.3	76.8 ± 15.7	78.4 ± 17.6	81.4 ± 18.4	0.009	0.17
24 h Urinary excretion						
Na/K ratio (mmol/mmol)	4.3 ± 1.1	4.1 ± 1.5	4.0 ± 1.2	3.7 ± 1.2	0.03	−0.16
SFAs point						
*n*	77	81	27	139		
Median (IQR)	8.3 (7.5–8.5)	9.3 (9.0–9.5)	9.8 (9.8)	10.0 (10.0)		
Dietary intake estimated from 24 h Urinary excretion *^§^						
Sodium (mg/d)	4453 ± 1417	4727 ± 1540	4855 ± 1645	5139 ± 1562	0.01	0.13
Potassium (mg/d)	2230 ± 675	2472 ± 864	2674 ± 858	2466 ± 831	0.03	0.08
Protein (g/d)	72.8 ± 18.8	76.8 ± 17.4	78.8 ± 15.9	78.7 ± 18.4	0.30	0.04
24 h Urinary excretion						
Na/K ratio (mmol/mmol)	4.0 ± 1.0	3.8 ± 1.1	3.7 ± 1.2	4.2 ± 1.4	0.33	0.03
Sodium point						
*n*	76	81	74	93		
Median (IQR)	6.0 (5.3–6.5)	7.4 (7.1–7.6)	8.3 (8.0–8.4)	9.1 (8.8–9.4)		
Dietary intake estimated from 24 h Urinary excretion *^§^						
Sodium (mg/d)	5575 ± 1893	4991 ± 1494	4670 ± 1377	4278 ± 1120	<0.001	−0.26
Potassium (mg/d)	2580 ± 922	2487 ± 724	2287 ± 870	2372 ± 732	0.30	−0.06
Protein (g/d)	78.4 ± 17.8	78.1 ± 16.4	75.0 ± 18.9	75.9 ± 19.2	0.64	−0.03
24 h Urinary excretion						
Na/K ratio (mmol/mmol)	4.3 ± 1.3	4.0 ± 1.1	4.2 ± 1.4	3.6 ± 1.1	0.002	−0.18

ANPS-Day, the Ajinomoto Group Nutrient Profiling System for Japanese Overall Diet per day; Q, quartile; SFAs, saturated fatty acids; Na/K ratio, sodium-to-potassium ratio; SD, standard deviation; IQR, inter-quartile range. * Values are presented as Mean ± SD. **^†^** Trend of association was examined using a multivariable linear regression model adjusted for age and sex, with the median value of the ANPS-Day score in each quartile modelled as a continuous variable. **^‡^** Partial Spearman correlation coefficients adjusted for age and sex were calculated. ^§^ Dietary intake estimated from 24 h urinary excretion was calculated as follows: sodium intake (mg/d) = 24 h urinary sodium excretion (mg/d)/0.86; potassium intake (mg/d) = 24 h urinary potassium excretion (mg/d)/0.77; and protein intake (g/d) = 24 h urinary urea-N (g/d)/(0.85 × 0.81) × 6.25.

## Data Availability

The dietary intake and 24 h urinary biomarker datasets analyzed during the current study are available on reasonable request from the corresponding author due to ethical and privacy restrictions.
